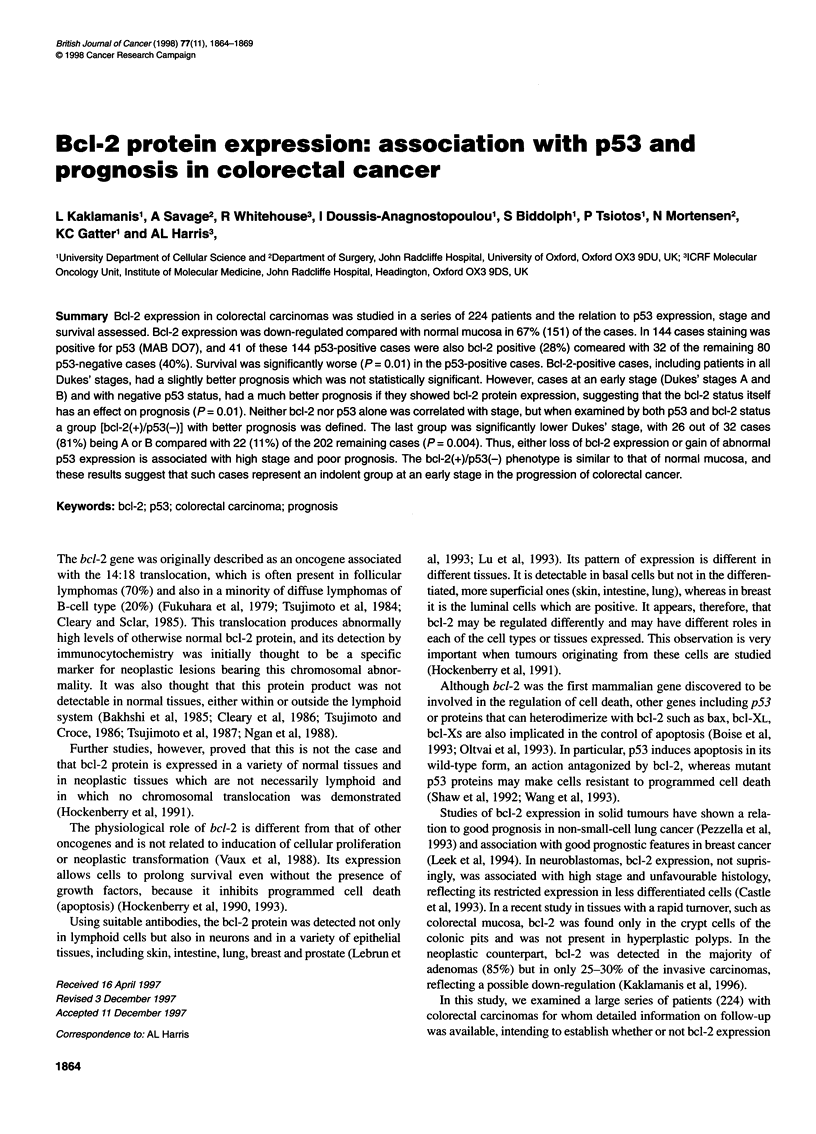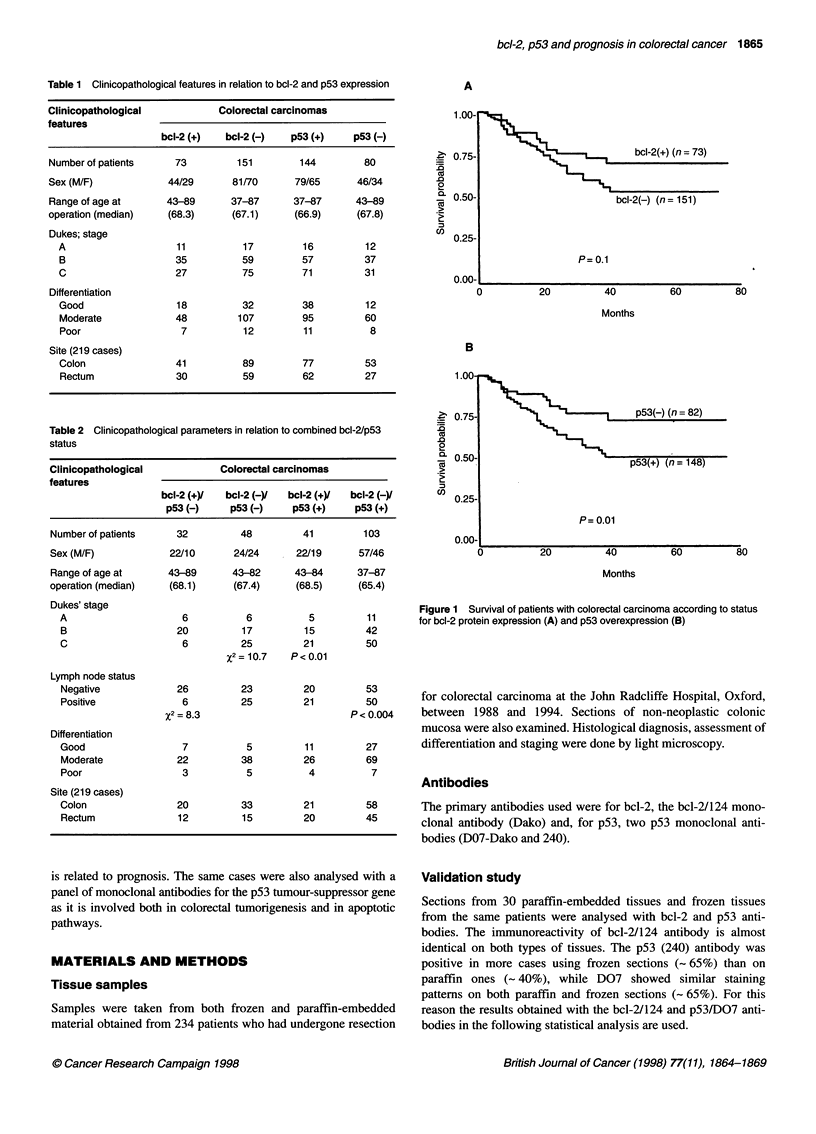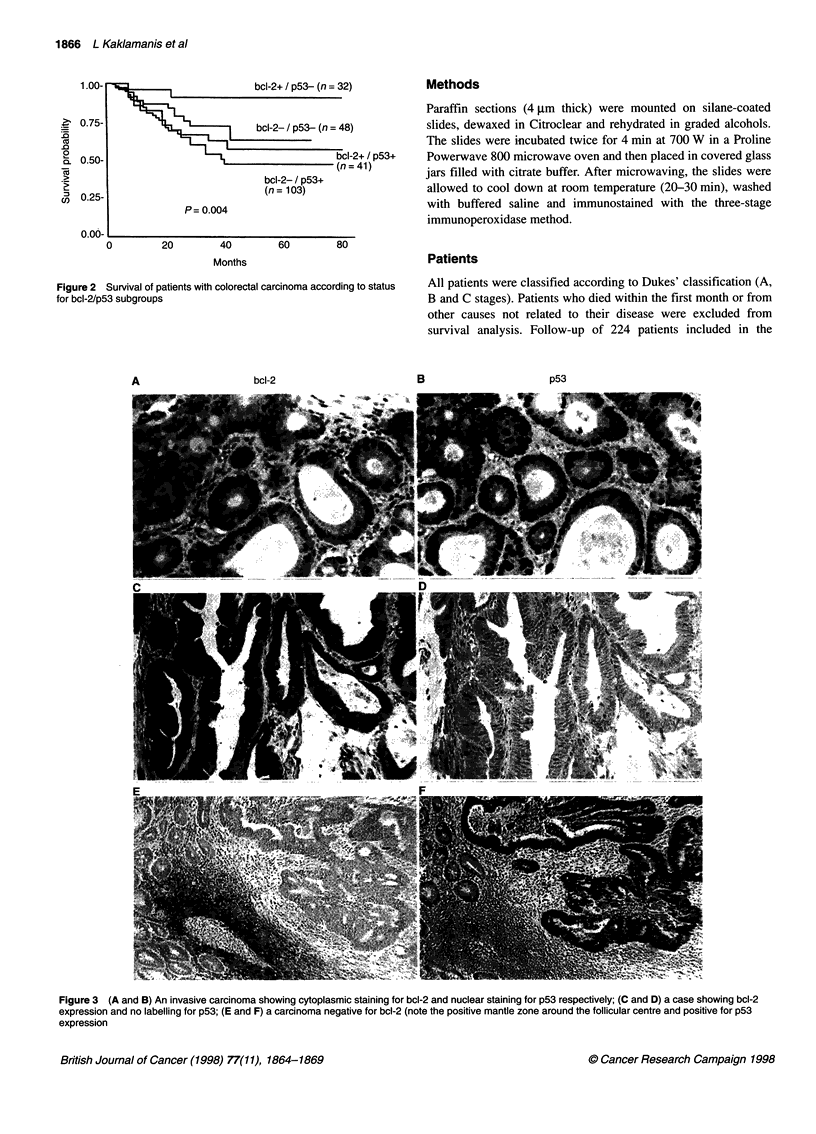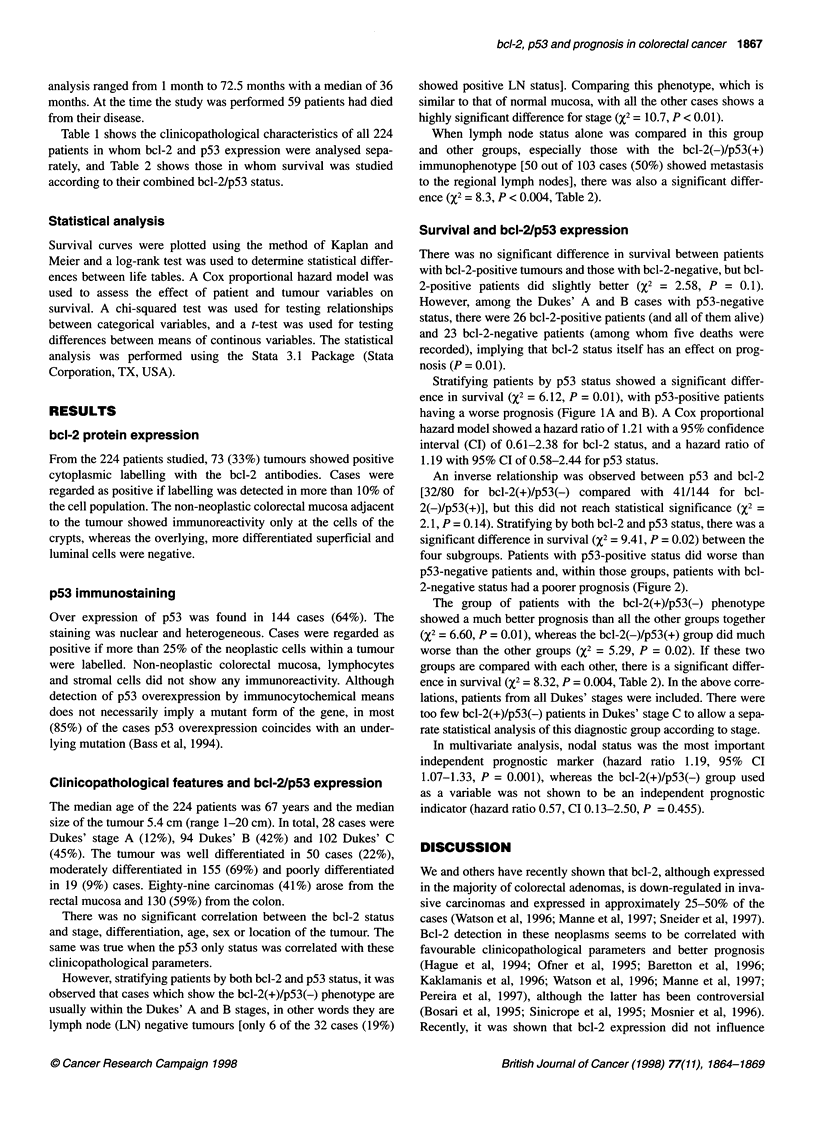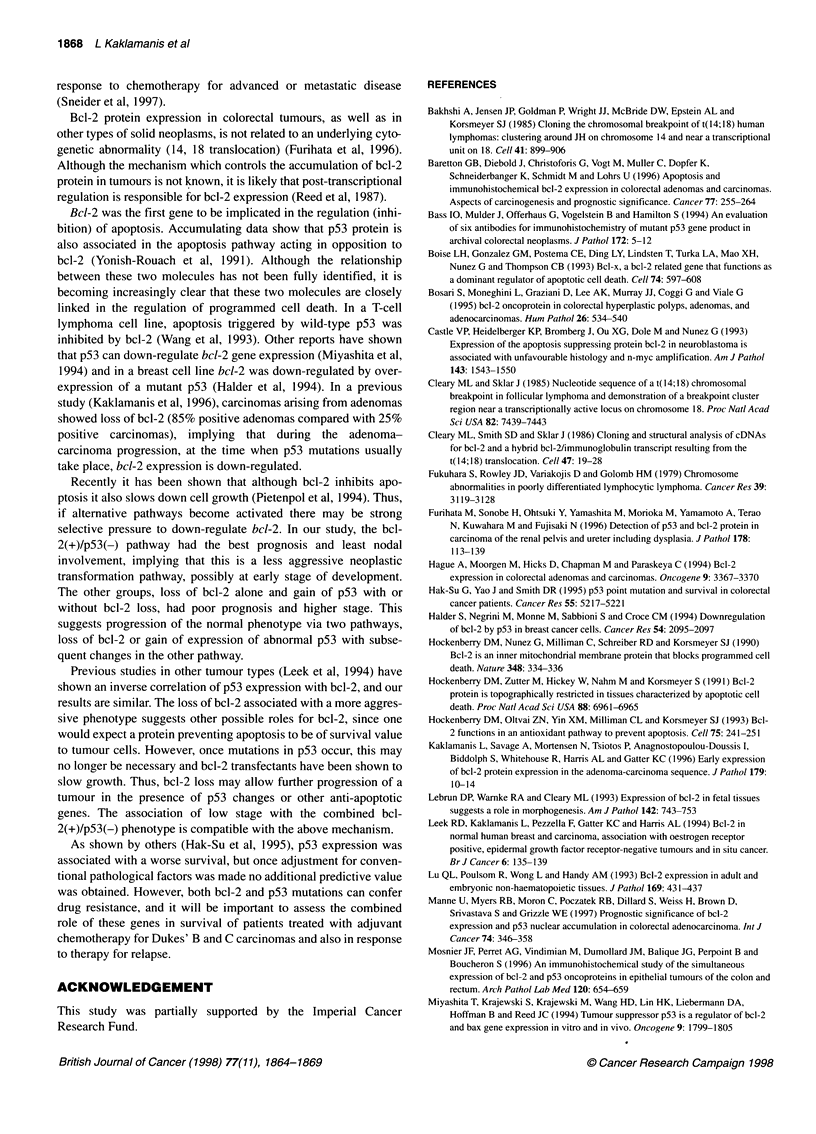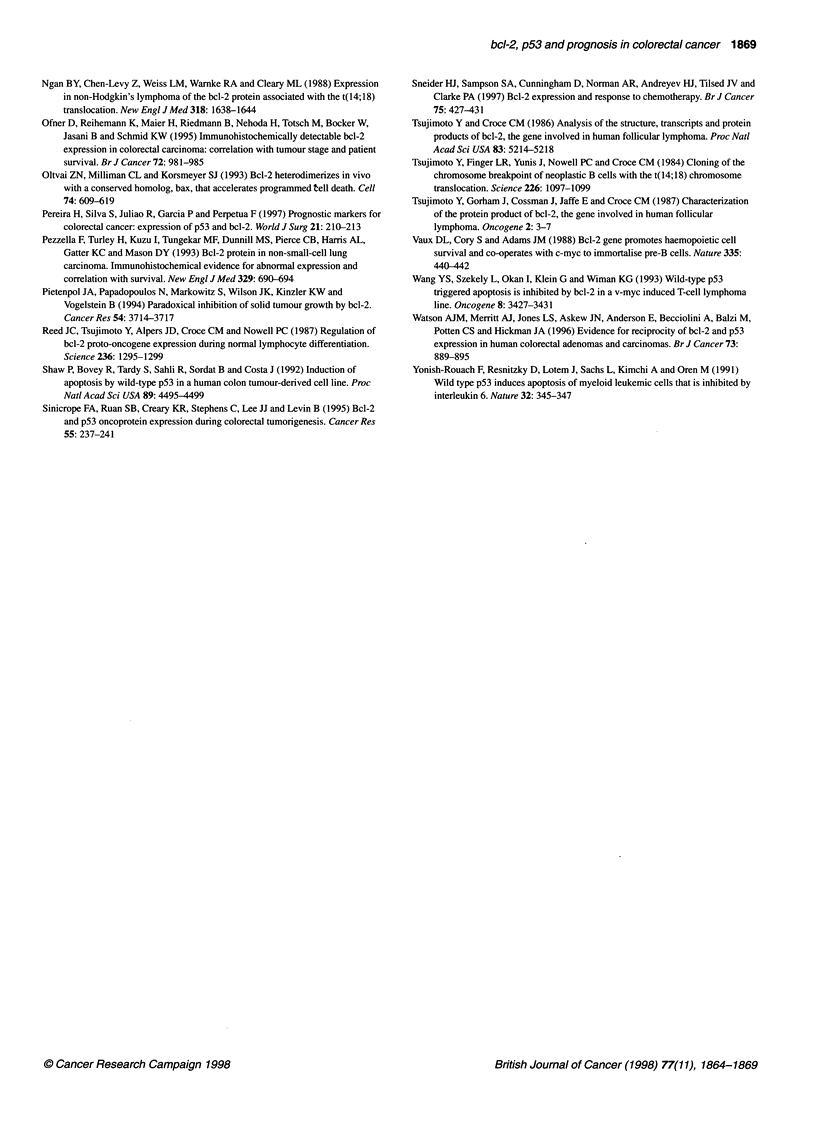# Bcl-2 protein expression: association with p53 and prognosis in colorectal cancer.

**DOI:** 10.1038/bjc.1998.310

**Published:** 1998-06

**Authors:** L. Kaklamanis, A. Savage, R. Whitehouse, I. Doussis-Anagnostopoulou, S. Biddolph, P. Tsiotos, N. Mortensen, K. C. Gatter, A. L. Harris

**Affiliations:** University Department of Cellular Science, John Radcliffe Hospital, University of Oxford, UK.

## Abstract

**Images:**


British Journal of Cancer (1998) 77(11), 1864–1869
© 1998 Cancer Research Campaign

### Bcl-2 protein expression: association with p53 and prognosis in colorectal cancer

L Kaklamanis[1], A Savage[2], R Whitehouse[3], I Doussis-Anagnostopoulou[1], S Biddolph[1], P Tsiotos[1], N Mortensen[2], KC Gatter[1] and AL Harris[3],

[1]University Department of Cellular Science and [2]Department of Surgery, John Radcliffe Hospital, University of Oxford, Oxford OX3 9DU, UK; [3]ICRF Molecular Oncology Unit, Institute of Molecular Medicine, John Radcliffe Hospital, Headington, Oxford OX3 9DS, UK

**Summary** Bcl-2 expression in colorectal carcinomas was studied in a series of 224 patients and the relation to p53 expression, stage and survival assessed. Bcl-2 expression was down-regulated compared with normal mucosa in 67% (151) of the cases. In 144 cases staining was positive for p53 (MAB DO7), and 41 of these 144 p53-positive cases were also bcl-2 positive (28%) comeared with 32 of the remaining 80 p53-negative cases (40%). Survival was significantly worse ($P = 0.01$) in the p53-positive cases. Bcl-2-positive cases, including patients in all Dukes' stages, had a slightly better prognosis which was not statistically significant. However, cases at an early stage (Dukes' stages A and B) and with negative p53 status, had a much better prognosis if they showed bcl-2 protein expression, suggesting that the bcl-2 status itself has an effect on prognosis ($P = 0.01$). Neither bcl-2 nor p53 alone was correlated with stage, but when examined by both p53 and bcl-2 status a group [bcl-2(+)/p53(−)] with better prognosis was defined. The last group was significantly lower Dukes' stage, with 26 out of 32 cases (81%) being A or B compared with 22 (11%) of the 202 remaining cases ($P = 0.004$). Thus, either loss of bcl-2 expression or gain of abnormal p53 expression is associated with high stage and poor prognosis. The bcl-2(+)/p53(−) phenotype is similar to that of normal mucosa, and these results suggest that such cases represent an indolent group at an early stage in the progression of colorectal cancer.

**Keywords:** bcl-2; p53; colorectal carcinoma; prognosis

The *bcl*-2 gene was originally described as an oncogene associated with the 14:18 translocation, which is often present in follicular lymphomas (70%) and also in a minority of diffuse lymphomas of B-cell type (20%) (Fukuhara et al, 1979; Tsujimoto et al, 1984; Cleary and Sclar, 1985). This translocation produces abnormally high levels of otherwise normal bcl-2 protein, and its detection by immunocytochemistry was initially thought to be a specific marker for neoplastic lesions bearing this chromosomal abnormality. It was also thought that this protein product was not detectable in normal tissues, either within or outside the lymphoid system (Bakhshi et al, 1985; Cleary et al, 1986; Tsujimoto and Croce, 1986; Tsujimoto et al, 1987; Ngan et al, 1988).

Further studies, however, proved that this is not the case and that bcl-2 protein is expressed in a variety of normal tissues and in neoplastic tissues which are not necessarily lymphoid and in which no chromosomal translocation was demonstrated (Hockenberry et al, 1991).

The physiological role of *bcl*-2 is different from that of other oncogenes and is not related to inducation of cellular proliferation or neoplastic transformation (Vaux et al, 1988). Its expression allows cells to prolong survival even without the presence of growth factors, because it inhibits programmed cell death (apoptosis) (Hockenberry et al, 1990, 1993).

Using suitable antibodies, the bcl-2 protein was detected not only in lymphoid cells but also in neurons and in a variety of epithelial tissues, including skin, intestine, lung, breast and prostate (Lebrun et al, 1993; Lu et al, 1993). Its pattern of expression is different in different tissues. It is detectable in basal cells but not in the differentiated, more superficial ones (skin, intestine, lung), whereas in breast it is the luminal cells which are positive. It appears, therefore, that bcl-2 may be regulated differently and may have different roles in each of the cell types or tissues expressed. This observation is very important when tumours originating from these cells are studied (Hockenberry et al, 1991).

Although *bcl*-2 was the first mammalian gene discovered to be involved in the regulation of cell death, other genes including *p53* or proteins that can heterodimerize with bcl-2 such as bax, bcl-XL, bcl-Xs are also implicated in the control of apoptosis (Boise et al, 1993; Oltvai et al, 1993). In particular, p53 induces apoptosis in its wild-type form, an action antagonized by bcl-2, whereas mutant p53 proteins may make cells resistant to programmed cell death (Shaw et al, 1992; Wang et al, 1993).

Studies of bcl-2 expression in solid tumours have shown a relation to good prognosis in non-small-cell lung cancer (Pezzella et al, 1993) and association with good prognostic features in breast cancer (Leek et al, 1994). In neuroblastomas, bcl-2 expression, not suprisingly, was associated with high stage and unfavourable histology, reflecting its restricted expression in less differentiated cells (Castle et al, 1993). In a recent study in tissues with a rapid turnover, such as colorectal mucosa, bcl-2 was found only in the crypt cells of the colonic pits and was not present in hyperplastic polyps. In the neoplastic counterpart, bcl-2 was detected in the majority of adenomas (85%) but in only 25–30% of the invasive carcinomas, reflecting a possible down-regulation (Kaklamanis et al, 1996).

In this study, we examined a large series of patients (224) with colorectal carcinomas for whom detailed information on follow-up was available, intending to establish whether or not bcl-2 expression

*Received 16 April 1997*
*Revised 3 December 1997*
*Accepted 11 December 1997*

*Correspondence to:* AL Harris

**Table 1** Clinicopathological features in relation to bcl-2 and p53 expression

| Clinicopathological features | Colorectal carcinomas | | | |
|---|---|---|---|---|
| | bcl-2 (+) | bcl-2 (–) | p53 (+) | p53 (–) |
| Number of patients | 73 | 151 | 144 | 80 |
| Sex (M/F) | 44/29 | 81/70 | 79/65 | 46/34 |
| Range of age at operation (median) | 43–89 (68.3) | 37–87 (67.1) | 37–87 (66.9) | 43–89 (67.8) |
| Dukes; stage | | | | |
| A | 11 | 17 | 16 | 12 |
| B | 35 | 59 | 57 | 37 |
| C | 27 | 75 | 71 | 31 |
| Differentiation | | | | |
| Good | 18 | 32 | 38 | 12 |
| Moderate | 48 | 107 | 95 | 60 |
| Poor | 7 | 12 | 11 | 8 |
| Site (219 cases) | | | | |
| Colon | 41 | 89 | 77 | 53 |
| Rectum | 30 | 59 | 62 | 27 |

**Table 2** Clinicopathological parameters in relation to combined bcl-2/p53 status

| Clinicopathological features | Colorectal carcinomas | | | |
|---|---|---|---|---|
| | bcl-2 (+)/ p53 (–) | bcl-2 (–)/ p53 (–) | bcl-2 (+)/ p53 (+) | bcl-2 (–)/ p53 (+) |
| Number of patients | 32 | 48 | 41 | 103 |
| Sex (M/F) | 22/10 | 24/24 | 22/19 | 57/46 |
| Range of age at operation (median) | 43–89 (68.1) | 43–82 (67.4) | 43–84 (68.5) | 37–87 (65.4) |
| Dukes' stage | | | | |
| A | 6 | 6 | 5 | 11 |
| B | 20 | 17 | 15 | 42 |
| C | 6 | 25 | 21 | 50 |
| | | $\chi^2 = 10.7$ | $P < 0.01$ | |
| Lymph node status | | | | |
| Negative | 26 | 23 | 20 | 53 |
| Positive | 6 | 25 | 21 | 50 |
| | $\chi^2 = 8.3$ | | | $P < 0.004$ |
| Differentiation | | | | |
| Good | 7 | 5 | 11 | 27 |
| Moderate | 22 | 38 | 26 | 69 |
| Poor | 3 | 5 | 4 | 7 |
| Site (219 cases) | | | | |
| Colon | 20 | 33 | 21 | 58 |
| Rectum | 12 | 15 | 20 | 45 |

is related to prognosis. The same cases were also analysed with a panel of monoclonal antibodies for the p53 tumour-suppressor gene as it is involved both in colorectal tumorigenesis and in apoptotic pathways.

### MATERIALS AND METHODS

### Tissue samples

Samples were taken from both frozen and paraffin-embedded material obtained from 234 patients who had undergone resection for colorectal carcinoma at the John Radcliffe Hospital, Oxford, between 1988 and 1994. Sections of non-neoplastic colonic mucosa were also examined. Histological diagnosis, assessment of differentiation and staging were done by light microscopy.

**Figure 1** Survival of patients with colorectal carcinoma according to status for bcl-2 protein expression (**A**) and p53 overexpression (**B**)

### Antibodies

The primary antibodies used were for bcl-2, the bcl-2/124 monoclonal antibody (Dako) and, for p53, two p53 monoclonal antibodies (D07-Dako and 240).

### Validation study

Sections from 30 paraffin-embedded tissues and frozen tissues from the same patients were analysed with bcl-2 and p53 antibodies. The immunoreactivity of bcl-2/124 antibody is almost identical on both types of tissues. The p53 (240) antibody was positive in more cases using frozen sections (~ 65%) than on paraffin ones (~ 40%), while DO7 showed similar staining patterns on both paraffin and frozen sections (~ 65%). For this reason the results obtained with the bcl-2/124 and p53/DO7 antibodies in the following statistical analysis are used.

© Cancer Research Campaign 1998

**Figure 2** Survival of patients with colorectal carcinoma according to status for bcl-2/p53 subgroups

### Methods

Paraffin sections (4 μm thick) were mounted on silane-coated slides, dewaxed in Citroclear and rehydrated in graded alcohols. The slides were incubated twice for 4 min at 700 W in a Proline Powerwave 800 microwave oven and then placed in covered glass jars filled with citrate buffer. After microwaving, the slides were allowed to cool down at room temperature (20–30 min), washed with buffered saline and immunostained with the three-stage immunoperoxidase method.

### Patients

All patients were classified according to Dukes' classification (A, B and C stages). Patients who died within the first month or from other causes not related to their disease were excluded from survival analysis. Follow-up of 224 patients included in the

**Figure 3** (**A** and **B**) An invasive carcinoma showing cytoplasmic staining for bcl-2 and nuclear staining for p53 respectively; (**C** and **D**) a case showing bcl-2 expression and no labelling for p53; (**E** and **F**) a carcinoma negative for bcl-2 (note the positive mantle zone around the follicular centre and positive for p53 expression

© Cancer Research Campaign 1998

analysis ranged from 1 month to 72.5 months with a median of 36 months. At the time the study was performed 59 patients had died from their disease.

Table 1 shows the clinicopathological characteristics of all 224 patients in whom bcl-2 and p53 expression were analysed separately, and Table 2 shows those in whom survival was studied according to their combined bcl-2/p53 status.

### Statistical analysis

Survival curves were plotted using the method of Kaplan and Meier and a log-rank test was used to determine statistical differences between life tables. A Cox proportional hazard model was used to assess the effect of patient and tumour variables on survival. A chi-squared test was used for testing relationships between categorical variables, and a *t*-test was used for testing differences between means of continous variables. The statistical analysis was performed using the Stata 3.1 Package (Stata Corporation, TX, USA).

### RESULTS

### bcl-2 protein expression

From the 224 patients studied, 73 (33%) tumours showed positive cytoplasmic labelling with the bcl-2 antibodies. Cases were regarded as positive if labelling was detected in more than 10% of the cell population. The non-neoplastic colorectal mucosa adjacent to the tumour showed immunoreactivity only at the cells of the crypts, whereas the overlying, more differentiated superficial and luminal cells were negative.

### p53 immunostaining

Over expression of p53 was found in 144 cases (64%). The staining was nuclear and heterogeneous. Cases were regarded as positive if more than 25% of the neoplastic cells within a tumour were labelled. Non-neoplastic colorectal mucosa, lymphocytes and stromal cells did not show any immunoreactivity. Although detection of p53 overexpression by immunocytochemical means does not necessarily imply a mutant form of the gene, in most (85%) of the cases p53 overexpression coincides with an underlying mutation (Bass et al, 1994).

### Clinicopathological features and bcl-2/p53 expression

The median age of the 224 patients was 67 years and the median size of the tumour 5.4 cm (range 1–20 cm). In total, 28 cases were Dukes' stage A (12%), 94 Dukes' B (42%) and 102 Dukes' C (45%). The tumour was well differentiated in 50 cases (22%), moderately differentiated in 155 (69%) and poorly differentiated in 19 (9%) cases. Eighty-nine carcinomas (41%) arose from the rectal mucosa and 130 (59%) from the colon.

There was no significant correlation between the bcl-2 status and stage, differentiation, age, sex or location of the tumour. The same was true when the p53 only status was correlated with these clinicopathological parameters.

However, stratifying patients by both bcl-2 and p53 status, it was observed that cases which show the bcl-2(+)/p53(–) phenotype are usually within the Dukes' A and B stages, in other words they are lymph node (LN) negative tumours [only 6 of the 32 cases (19%) showed positive LN status]. Comparing this phenotype, which is similar to that of normal mucosa, with all the other cases shows a highly significant difference for stage ($\chi^2 = 10.7$, $P < 0.01$).

When lymph node status alone was compared in this group and other groups, especially those with the bcl-2(–)/p53(+) immunophenotype [50 out of 103 cases (50%) showed metastasis to the regional lymph nodes], there was also a significant difference ($\chi^2 = 8.3$, $P < 0.004$, Table 2).

### Survival and bcl-2/p53 expression

There was no significant difference in survival between patients with bcl-2-positive tumours and those with bcl-2-negative, but bcl-2-positive patients did slightly better ($\chi^2 = 2.58$, $P = 0.1$). However, among the Dukes' A and B cases with p53-negative status, there were 26 bcl-2-positive patients (and all of them alive) and 23 bcl-2-negative patients (among whom five deaths were recorded), implying that bcl-2 status itself has an effect on prognosis ($P = 0.01$).

Stratifying patients by p53 status showed a significant difference in survival ($\chi^2 = 6.12$, $P = 0.01$), with p53-positive patients having a worse prognosis (Figure 1A and B). A Cox proportional hazard model showed a hazard ratio of 1.21 with a 95% confidence interval (CI) of 0.61–2.38 for bcl-2 status, and a hazard ratio of 1.19 with 95% CI of 0.58–2.44 for p53 status.

An inverse relationship was observed between p53 and bcl-2 [32/80 for bcl-2(+)/p53(–) compared with 41/144 for bcl-2(–)/p53(+)], but this did not reach statistical significance ($\chi^2 = 2.1$, $P = 0.14$). Stratifying by both bcl-2 and p53 status, there was a significant difference in survival ($\chi^2 = 9.41$, $P = 0.02$) between the four subgroups. Patients with p53-positive status did worse than p53-negative patients and, within those groups, patients with bcl-2-negative status had a poorer prognosis (Figure 2).

The group of patients with the bcl-2(+)/p53(–) phenotype showed a much better prognosis than all the other groups together ($\chi^2 = 6.60$, $P = 0.01$), whereas the bcl-2(–)/p53(+) group did much worse than the other groups ($\chi^2 = 5.29$, $P = 0.02$). If these two groups are compared with each other, there is a significant difference in survival ($\chi^2 = 8.32$, $P = 0.004$, Table 2). In the above correlations, patients from all Dukes' stages were included. There were too few bcl-2(+)/p53(–) patients in Dukes' stage C to allow a separate statistical analysis of this diagnostic group according to stage.

In multivariate analysis, nodal status was the most important independent prognostic marker (hazard ratio 1.19, 95% CI 1.07–1.33, $P = 0.001$), whereas the bcl-2(+)/p53(–) group used as a variable was not shown to be an independent prognostic indicator (hazard ratio 0.57, CI 0.13–2.50, $P = 0.455$).

### DISCUSSION

We and others have recently shown that bcl-2, although expressed in the majority of colorectal adenomas, is down-regulated in invasive carcinomas and expressed in approximately 25–50% of the cases (Watson et al, 1996; Manne et al, 1997; Sneider et al, 1997). Bcl-2 detection in these neoplasms seems to be correlated with favourable clinicopathological parameters and better prognosis (Hague et al, 1994; Ofner et al, 1995; Baretton et al, 1996; Kaklamanis et al, 1996; Watson et al, 1996; Manne et al, 1997; Pereira et al, 1997), although the latter has been controversial (Bosari et al, 1995; Sinicrope et al, 1995; Mosnier et al, 1996). Recently, it was shown that bcl-2 expression did not influence

© Cancer Research Campaign 1998

response to chemotherapy for advanced or metastatic disease (Sneider et al, 1997).

Bcl-2 protein expression in colorectal tumours, as well as in other types of solid neoplasms, is not related to an underlying cytogenetic abnormality (14, 18 translocation) (Furihata et al, 1996). Although the mechanism which controls the accumulation of bcl-2 protein in tumours is not known, it is likely that post-transcriptional regulation is responsible for bcl-2 expression (Reed et al, 1987).

*Bcl*-2 was the first gene to be implicated in the regulation (inhibition) of apoptosis. Accumulating data show that p53 protein is also associated in the apoptosis pathway acting in opposition to bcl-2 (Yonish-Rouach et al, 1991). Although the relationship between these two molecules has not been fully identified, it is becoming increasingly clear that these two molecules are closely linked in the regulation of programmed cell death. In a T-cell lymphoma cell line, apoptosis triggered by wild-type p53 was inhibited by bcl-2 (Wang et al, 1993). Other reports have shown that p53 can down-regulate *bcl*-2 gene expression (Miyashita et al, 1994) and in a breast cell line *bcl*-2 was down-regulated by over-expression of a mutant p53 (Halder et al, 1994). In a previous study (Kaklamanis et al, 1996), carcinomas arising from adenomas showed loss of bcl-2 (85% positive adenomas compared with 25% positive carcinomas), implying that during the adenoma–carcinoma progression, at the time when p53 mutations usually take place, *bcl*-2 expression is down-regulated.

Recently it has been shown that although bcl-2 inhibits apoptosis it also slows down cell growth (Pietenpol et al, 1994). Thus, if alternative pathways become activated there may be strong selective pressure to down-regulate *bcl*-2. In our study, the bcl-2(+)/p53(–) pathway had the best prognosis and least nodal involvement, implying that this is a less aggressive neoplastic transformation pathway, possibly at early stage of development. The other groups, loss of bcl-2 alone and gain of p53 with or without bcl-2 loss, had poor prognosis and higher stage. This suggests progression of the normal phenotype via two pathways, loss of bcl-2 or gain of expression of abnormal p53 with subsequent changes in the other pathway.

Previous studies in other tumour types (Leek et al, 1994) have shown an inverse correlation of p53 expression with bcl-2, and our results are similar. The loss of bcl-2 associated with a more aggressive phenotype suggests other possible roles for bcl-2, since one would expect a protein preventing apoptosis to be of survival value to tumour cells. However, once mutations in p53 occur, this may no longer be necessary and bcl-2 transfectants have been shown to slow growth. Thus, bcl-2 loss may allow further progression of a tumour in the presence of p53 changes or other anti-apoptotic genes. The association of low stage with the combined bcl-2(+)/p53(–) phenotype is compatible with the above mechanism.

As shown by others (Hak-Su et al, 1995), p53 expression was associated with a worse survival, but once adjustment for conventional pathological factors was made no additional predictive value was obtained. However, both bcl-2 and p53 mutations can confer drug resistance, and it will be important to assess the combined role of these genes in survival of patients treated with adjuvant chemotherapy for Dukes' B and C carcinomas and also in response to therapy for relapse.

### ACKNOWLEDGEMENT

This study was partially supported by the Imperial Cancer Research Fund.

### REFERENCES

Bakhshi A, Jensen JP, Goldman P, Wright JJ, McBride DW, Epstein AL and Korsmeyer SJ (1985) Cloning the chromosomal breakpoint of t(14;18) human lymphomas: clustering around JH on chromosome 14 and near a transcriptional unit on 18. *Cell* **41**: 899–906

Baretton GB, Diebold J, Christoforis G, Vogt M, Muller C, Dopfer K, Schneiderbanger K, Schmidt M and Lohrs U (1996) Apoptosis and immunohistochemical bcl-2 expression in colorectal adenomas and carcinomas. Aspects of carcinogenesis and prognostic significance. *Cancer* **77**: 255–264

Bass IO, Mulder J, Offerhaus G, Vogelstein B and Hamilton S (1994) An evaluation of six antibodies for immunohistochemistry of mutant p53 gene product in archival colorectal neoplasms. *J Pathol* **172**: 5–12

Boise LH, Gonzalez GM, Postema CE, Ding LY, Lindsten T, Turka LA, Mao XH, Nunez G and Thompson CB (1993) Bcl-x, a bcl-2 related gene that functions as a dominant regulator of apoptotic cell death. *Cell* **74**: 597–608

Bosari S, Moneghini L, Graziani D, Lee AK, Murray JJ, Coggi G and Viale G (1995) bcl-2 oncoprotein in colorectal hyperplastic polyps, adenomas, and adenocarcinomas. *Hum Pathol* **26**: 534–540

Castle VP, Heidelberger KP, Bromberg J, Ou XG, Dole M and Nunez G (1993) Expression of the apoptosis suppressing protein bcl-2 in neuroblastoma is associated with unfavourable histology and n-myc amplification. *Am J Pathol* **143**: 1543–1550

Cleary ML and Sklar J (1985) Nucleotide sequence of a t(14;18) chromosomal breakpoint in follicular lymphoma and demonstration of a breakpoint cluster region near a transcriptionally active locus on chromosome 18. *Proc Natl Acad Sci USA* **82**: 7439–7443

Cleary ML, Smith SD and Sklar J (1986) Cloning and structural analysis of cDNAs for bcl-2 and a hybrid bcl-2/immunoglobulin transcript resulting from the t(14;18) translocation. *Cell* **47**: 19–28

Fukuhara S, Rowley JD, Variakojis D and Golomb HM (1979) Chromosome abnormalities in poorly differentiated lymphocytic lymphoma. *Cancer Res* **39**: 3119–3128

Furihata M, Sonobe H, Ohtsuki Y, Yamashita M, Morioka M, Yamamoto A, Terao N, Kuwahara M and Fujisaki N (1996) Detection of p53 and bcl-2 protein in carcinoma of the renal pelvis and ureter including dysplasia. *J Pathol* **178**: 113–139

Hague A, Moorgen M, Hicks D, Chapman M and Paraskeya C (1994) Bcl-2 expression in colorectal adenomas and carcinomas. *Oncogene* **9**: 3367–3370

Hak-Su G, Yao J and Smith DR (1995) p53 point mutation and survival in colorectal cancer patients. *Cancer Res* **55**: 5217–5221

Halder S, Negrini M, Monne M, Sabbioni S and Croce CM (1994) Downregulation of bcl-2 by p53 in breast cancer cells. *Cancer Res* **54**: 2095–2097

Hockenberry DM, Nunez G, Milliman C, Schreiber RD and Korsmeyer SJ (1990) Bcl-2 is an inner mitochondrial membrane protein that blocks programmed cell death. *Nature* **348**: 334–336

Hockenberry DM, Zutter M, Hickey W, Nahm M and Korsmeyer S (1991) Bcl-2 protein is topographically restricted in tissues characterized by apoptotic cell death. *Proc Natl Acad Sci USA* **88**: 6961–6965

Hockenberry DM, Oltvai ZN, Yin XM, Milliman CL and Korsmeyer SJ (1993) Bcl-2 functions in an antioxidant pathway to prevent apoptosis. *Cell* **75**: 241–251

Kaklamanis L, Savage A, Mortensen N, Tsiotos P, Anagnostopoulou-Doussis I, Biddolph S, Whitehouse R, Harris AL and Gatter KC (1996) Early expression of bcl-2 protein expression in the adenoma-carcinoma sequence. *J Pathol* **179**: 10–14

Lebrun DP, Warnke RA and Cleary ML (1993) Expression of bcl-2 in fetal tissues suggests a role in morphogenesis. *Am J Pathol* **142**: 743–753

Leek RD, Kaklamanis L, Pezzella F, Gatter KC and Harris AL (1994) Bcl-2 in normal human breast and carcinoma, association with oestrogen receptor positive, epidermal growth factor receptor-negative tumours and in situ cancer. *Br J Cancer* **6**: 135–139

Lu QL, Poulsom R, Wong L and Handy AM (1993) Bcl-2 expression in adult and embryonic non-haematopoietic tissues. *J Pathol* **169**: 431–437

Manne U, Myers RB, Moron C, Poczatek RB, Dillard S, Weiss H, Brown D, Srivastava S and Grizzle WE (1997) Prognostic significance of bcl-2 expression and p53 nuclear accumulation in colorectal adenocarcinoma. *Int J Cancer* **74**: 346–358

Mosnier JF, Perret AG, Vindimian M, Dumollard JM, Balique JG, Perpoint B and Boucheron S (1996) An immunohistochemical study of the simultaneous expression of bcl-2 and p53 oncoproteins in epithelial tumours of the colon and rectum. *Arch Pathol Lab Med* **120**: 654–659

Miyashita T, Krajewski S, Krajewski M, Wang HD, Lin HK, Liebermann DA, Hoffman B and Reed JC (1994) Tumour suppressor p53 is a regulator of bcl-2 and bax gene expression in vitro and in vivo. *Oncogene* **9**: 1799–1805

© Cancer Research Campaign 1998

Ngan BY, Chen-Levy Z, Weiss LM, Warnke RA and Cleary ML (1988) Expression in non-Hodgkin's lymphoma of the bcl-2 protein associated with the t(14;18) translocation. *New Engl J Med* **318**: 1638–1644

Ofner D, Reihemann K, Maier H, Riedmann B, Nehoda H, Totsch M, Bocker W, Jasani B and Schmid KW (1995) Immunohistochemically detectable bcl-2 expression in colorectal carcinoma: correlation with tumour stage and patient survival. *Br J Cancer* **72**: 981–985

Oltvai ZN, Milliman CL and Korsmeyer SJ (1993) Bcl-2 heterodimerizes in vivo with a conserved homolog, bax, that accelerates programmed cell death. *Cell* **74**: 609–619

Pereira H, Silva S, Juliao R, Garcia P and Perpetua F (1997) Prognostic markers for colorectal cancer: expression of p53 and bcl-2. *World J Surg* **21**: 210–213

Pezzella F, Turley H, Kuzu I, Tungekar MF, Dunnill MS, Pierce CB, Harris AL, Gatter KC and Mason DY (1993) Bcl-2 protein in non-small-cell lung carcinoma. Immunohistochemical evidence for abnormal expression and correlation with survival. *New Engl J Med* **329**: 690–694

Pietenpol JA, Papadopoulos N, Markowitz S, Wilson JK, Kinzler KW and Vogelstein B (1994) Paradoxical inhibition of solid tumour growth by bcl-2. *Cancer Res* **54**: 3714–3717

Reed JC, Tsujimoto Y, Alpers JD, Croce CM and Nowell PC (1987) Regulation of bcl-2 proto-oncogene expression during normal lymphocyte differentiation. *Science* **236**: 1295–1299

Shaw P, Bovey R, Tardy S, Sahli R, Sordat B and Costa J (1992) Induction of apoptosis by wild-type p53 in a human colon tumour-derived cell line. *Proc Natl Acad Sci USA* **89**: 4495–4499

Sinicrope FA, Ruan SB, Creary KR, Stephens C, Lee JJ and Levin B (1995) Bcl-2 and p53 oncoprotein expression during colorectal tumorigenesis. *Cancer Res* **55**: 237–241

Sneider HJ, Sampson SA, Cunningham D, Norman AR, Andreyev HJ, Tilsed JV and Clarke PA (1997) Bcl-2 expression and response to chemotherapy. *Br J Cancer* **75**: 427–431

Tsujimoto Y and Croce CM (1986) Analysis of the structure, transcripts and protein products of bcl-2, the gene involved in human follicular lymphoma. *Proc Natl Acad Sci USA* **83**: 5214–5218

Tsujimoto Y, Finger LR, Yunis J, Nowell PC and Croce CM (1984) Cloning of the chromosome breakpoint of neoplastic B cells with the t(14;18) chromosome translocation. *Science* **226**: 1097–1099

Tsujimoto Y, Gorham J, Cossman J, Jaffe E and Croce CM (1987) Characterization of the protein product of bcl-2, the gene involved in human follicular lymphoma. *Oncogene* **2**: 3–7

Vaux DL, Cory S and Adams JM (1988) Bcl-2 gene promotes haemopoietic cell survival and co-operates with c-myc to immortalise pre-B cells. *Nature* **335**: 440–442

Wang YS, Szekely L, Okan I, Klein G and Wiman KG (1993) Wild-type p53 triggered apoptosis is inhibited by bcl-2 in a v-myc induced T-cell lymphoma line. *Oncogene* **8**: 3427–3431

Watson AJM, Merritt AJ, Jones LS, Askew JN, Anderson E, Becciolini A, Balzi M, Potten CS and Hickman JA (1996) Evidence for reciprocity of bcl-2 and p53 expression in human colorectal adenomas and carcinomas. *Br J Cancer* **73**: 889–895

Yonish-Rouach F, Resnitzky D, Lotem J, Sachs L, Kimchi A and Oren M (1991) Wild type p53 induces apoptosis of myeloid leukemic cells that is inhibited by interleukin 6. *Nature* **32**: 345–347

© Cancer Research Campaign 1998